# Relationship between health literacy and generalized anxiety disorder during the COVID-19 pandemic in Khuzestan province, Iran

**DOI:** 10.3389/fpsyg.2023.1294562

**Published:** 2024-01-12

**Authors:** Amirreza Dadgarinejad, Nargess Nazarihermoshi, Negar Hematichegeni, Mahta Jazaiery, Shabnam Yousefishad, Hashem Mohammadian, Mehdi Sayyah, Maryam Dastoorpoor, Maria Cheraghi

**Affiliations:** ^1^School of Dentistry, Student Research Committee, Ahvaz Jundishapur University of Medical Sciences, Ahvaz, Iran; ^2^Health in Emergency and Disaster Research Center, University of Social Welfare and Rehabilitation Sciences, Tehran, Iran; ^3^School of Medicine, Ahvaz Jundishapur University of Medical Sciences, Ahvaz, Iran; ^4^Department of Public Health, School of Health, Ahvaz Jundishapur University of Medical Sciences, Ahvaz, Iran; ^5^Department of Health Promotion and Education, School of Health, Ahvaz Jundishapur University of Medical Sciences, Ahvaz, Iran; ^6^Department of Psychiatry, Educational Development Center, Ahvaz Jundishapur University of Medical Sciences, Ahvaz, Iran; ^7^Department of Biostatics and Epidemiology, School of Health, Ahvaz Jundishapur University of Medical Sciences, Ahvaz, Iran; ^8^Social Determinants of Health Research Center, Department of Public Health, School of Health, Ahvaz Jundishapur University of Medical Sciences, Ahvaz, Iran

**Keywords:** health literacy, generalized anxiety disorder, COVID-19, Iran, pandemic

## Abstract

**Introduction:**

During the COVID-19 pandemic, many changes occurred in various cultural, social, and economic fields, leading to the creation of psychological effects, especially anxiety, in the community. Fear and anxiety about emerging diseases (COVID-19) and less participation in preventive behaviors reduce individual resistance and ultimately lower one’s quality of life. Therefore, we aimed to investigate the relationship between health literacy and generalized anxiety disorder during the COVID-19 pandemic in Khuzestan province, Iran.

**Methods:**

This was a descriptive-analytical (cross-sectional) study conducted among participants aged 18–65 in Khuzestan province during the years 2020–2021 through online sampling. Due to the prevalence of COVID-19 and the impossibility of face-to-face communication, the questionnaire was designed on the Porseline Survey website and the questionnaire link was provided to participants through WhatsApp and Telegram. The data collection tool includes the Iranian Health Literacy Questionnaire, which includes 33 items, and the Generalized Anxiety Disorder-7 scale, which has 7 items based on a Likert scale. Data analysis was performed using STATA14 software and descriptive and analytical statistical tests at a significant level less than 0.05.

**Results:**

The mean (standard deviation) score for health literacy was 52.9 ± 9.3 and for generalized anxiety disorder was 5.2 ± 3.1. A significant negative correlation was found between the mean of anxiety disorder and health literacy (*p* < 0.05). The frequency of health literacy in individuals included 427 (37.8%) with inadequate health literacy, 628 (55.6%) with marginal health literacy, and 75 (6.6%) with sufficient health literacy. There was a statistically significant difference between the mean of generalized anxiety disorder among individuals with different levels of health literacy (*p* < 0.05).

**Conclusion:**

As the level of health literacy increases, the prevalence of generalized anxiety disorder caused by fear of COVID-19 decreases. Therefore, increasing awareness and health literacy about this virus, its transmission, and prevention methods is very effective in managing anxiety and stress caused by COVID-19. Paying attention to the issue of health literacy and generalized anxiety disorder, and promoting preventive behaviors can be effective tools for planners, health officials, and policymakers to promote health literacy for any type of disease.

## Introduction

The COVID-19 pandemic has affected millions of people worldwide and has caused significant health, social, and economic consequences. Iran, like many other countries, has experienced a high number of cases and deaths due to the virus. In addition to the physical health impact, the pandemic has also caused psychological distress, including death anxiety (Khademian et al., 2021; Zarei et al., 2021a,b; Jazaiery et al., 2022).

Health literacy (HL) is defined as the degree to which individuals have the capacity to obtain, process, and understand basic health information and services needed to make appropriate health decisions. According to the World Health Organization (WHO), health literacy is an important determinant of health outcomes and has a significant impact on individuals’ ability to access and use health services effectively (Health literacy in Healthy People 2030, 2021).

Studies have shown that enhancing HL can have a positive impact on resilience, health behavior, and well-being (Barsell et al., 2018). In the context of the COVID-19 pandemic, health literacy has become increasingly important as individuals seek accurate and timely information about the virus and its impact on their health. A study by Paakkari and Okan (2020) found that individuals with higher health literacy were more likely to engage in preventive behaviors and had a better understanding of the risks associated with COVID-19 (Paakkari and Okan, 2020). Low health literacy is associated with issues such as insufficient understanding of health information regarding fear of emerging diseases like COVID-19, less participation in preventive behaviors, delayed diagnosis of diseases (Schulz and Rapaport, 2005), inability to perform self-care skills (Von Wagner et al., 2007), and non-adherence to healthy lifestyle behaviors (Wolf et al., 2005).

Generalized anxiety disorder (GAD) is a mental health condition characterized by excessive and persistent worry and anxiety about everyday life events and activities. People with GAD may experience symptoms such as restlessness, fatigue, difficulty concentrating, irritability, muscle tension, and sleep disturbance. The exact causes of GAD are not fully understood, but it is believed to be a combination of genetic, biological, and environmental factors (Paulus et al., 2015). Generalized anxiety disorder is more prevalent among individuals with limited health literacy (Rowlands et al., 2013).

The COVID-19 pandemic has had a significant impact on mental health globally, including an increase in anxiety and stress levels. Several studies have suggested a potential association between COVID-19 and GAD. A study from China found that individuals who had been in quarantine during the COVID-19 outbreak had higher levels of anxiety and depression symptoms, including GAD symptoms, compared to those who had not been quarantined (Wang et al., 2020).

An Italian study during the COVID-19 pandemic found that individuals with pre-existing mental health conditions, including GAD, were at higher risk of experiencing severe psychological distress during the pandemic (Mazza et al., 2020).

The association between COVID-19 and GAD may be related to several factors, including fear of infection, uncertainty about the future, social isolation, financial stress, and other pandemic-related stressors. Additionally, the pandemic has disrupted usual routines and social support systems, which can exacerbate symptoms of GAD (Salari et al., 2020).

The relationship between health literacy and GAD during the COVID-19 pandemic is an important area of study. Health literacy refers to an individual’s ability to access, understand, and use health information to make informed decisions about their health (Paakkari and Okan, 2020). GAD, on the other hand, is a mental health condition characterized by excessive and persistent worry and anxiety (Paulus et al., 2015).

During the COVID-19 pandemic, individuals with higher health literacy may have better access to accurate and reliable health information, leading to a better understanding of the virus, preventive measures, and available healthcare resources. This increased understanding may help alleviate anxiety related to the pandemic (Vahedian-Azimi et al., 2020).

Conversely, individuals with lower health literacy may face challenges in accessing and understanding health information, which could contribute to heightened anxiety and uncertainty during the pandemic (Mohammadkhah et al., 2021).

However, it is important to note that the relationship between health literacy and GAD during COVID-19 is complex and influenced by various factors such as socioeconomic status, education level, cultural beliefs, and access to healthcare services. And there has been limited attention given to its relationship with generalized anxiety disorder, globally as well as in Iran. Several studies conducted in Iran have investigated the psychological link between generalized anxiety disorder and COVID-19 (Vahedian-Azimi et al., 2020), while only a few have explored the relationship between COVID-19 anxiety and health literacy (Mohammadkhah et al., 2021). Additionally, no such study has been conducted in the Khuzestan region which, with different races, cultures, and epidemiological factors, may have varying levels of health literacy that can impact one’s understanding and response to health information during the pandemic. Therefore, we designed this study to investigate the relationship between health literacy and generalized anxiety disorder levels among residents of Khuzestan province during the COVID-19 pandemic.

## Methods

### Study design and collect the data

This was a cross-sectional study conducted among 1,130 individuals aged 18–65 in Khuzestan province through online sampling from 2020–2021. The inclusion criteria for the study were willingness to participate, literacy, having a smartphone, and residing in Khuzestan province. Due to the prevalence of the COVID-19 disease and the impossibility of face-to-face communication, the questionnaire was designed on the Persian online survey platform named “Porseline” and the questionnaire link was made available to participants through social media online such as WhatsApp, Telegram, and Eitaa.

### Tools of study

We used two standard questionnaires as follows:

1. “Iranian Health Literacy Questionnaire.” The questionnaire consists of 33 items based on a five-point Likert scale (1: Always, 2: Mostly, 3: Sometimes, 4: Rarely, 5: Never). The questionnaire has five components, including access (items 1–6), reading skills (items 7–10), understanding (items 11–17), evaluation (items 18–20), and decision-making and application of information (items 21–33). The health literacy score ranges from 0 to 100 and is divided into four levels: inadequate (0–50), marginal (50.1–66), adequate (66.1–84), and excellent (84.1–100). In the present study, the adequate and excellent levels are considered as the adequate level (66.1–100). The Cronbach’s alpha coefficient for this questionnaire was reported to be 0.75 (Haghdoost et al., 2015).
2. The Generalized Anxiety Disorder-7 questionnaire consists of seven items based on a four-point Likert scale (1: Never, 2: Several days, 3: Nearly every day, 4: About half the days). The scores range from 7 to 21, and higher scores indicate higher anxiety levels. The Cronbach’s alpha coefficient for this standard questionnaire was reported to be 0.85 (Nayiniyan et al., 2011).

### Ethical considerations

This study was implemented with the support of the Social Determinant of Health Research Center (Reference No. SDH-9904), Deputy of Research in Ahvaz Jundishapur University of Medical Sciences with ethics committee number: IR.AJUMS.REC.1399.089.

### Statistical analysis

In this study, all statistical analyses were performed by using STATA version 14 software.

Descriptive statistics for frequency, percentage, mean, and standard deviation were performed.

And for analytical: we used independed *t*-test, one way ANOWA. The level of significance was less than 0.05.

## Results

The results of this study were obtained from 1,130 participants in the age groups under study. There were 40 individuals (3.5%) in the first age group, 522 individuals (46.2%) in the second age group, 525 individuals (46.5%) in the third age group, and 43 individuals (3.8%) in the fourth age group. In addition, there were 552 men (48.8%) and 578 women (51.2%). There were 875 married participants (77.4%). The majority of participants, 436 (38.6%), had a bachelor’s degree, and 255 had an associate degree, with the lowest percentage (22.7%). Regarding occupation, employed individuals had the highest frequency with 638 (56.4%), and unemployed individuals had the lowest frequency with 55 (4.9%) (Table 1).

**Table 1:** Relationship between demographic variables and health literacy and generalized anxiety disorder.

Variables	Subgroups	No. (%)	Health literacy	Value of *p*	General Anxiety Disorder	Value of *p*
			Mean (SD)		Mean (SD)	
Age	Less than 20 years	40 (3.5)	51.45 (12.47)	0.001	4.40 (3.20)	0.006
	20–40 years	522 (46.2)	51.39 (9.60)		5.55 (3.07)	
	More than 60 years	568 (50.3)	54.41 (8.70)		5.03 (3.22)	
Gender	Men	552 (48.8)	52.55 (9.35)	0.2	5.03 (3.24)	0.02
	Women	578 (51.2)	53.26 (9.43)		5.46 (3.08)	
Marital status	Single	255 (22.6)	51.90 (11.05)	0.05	4.89 (2.98)	0.03
	Married	875 (77.4)	53.21 (8.84)		5.36 (3.21)	
Level of education	School degree	233 (20.6)	52.16 (9.85)	0.01	5.54 (3.28)	0.2
	Undergraduate	518 (45.8)	52.44 (9.62)		5.25 (3.15)	
	Post graduate	379 (33.5)	54.01 (8.69)		5.08 (3.10)	
Employment	Unemployed	194 (17.2)	51.58 (11.03)	0.02	5.43 (3.15)	0.001
	Employee	171 (15.1)	52.88 (9.48)		5.94 (3.13)	
	Retired	638 (56.5)	52.92 (9.11)		5.15 (3.16)	
	Homemaker	127 (11.2)	54.92 (7.51)		4.56 (3.08)	

Table 1 presents the results of a study investigating the relationship between demographic variables (age, gender, marital status, level of education, and employment) and health literacy and GAD among the study population. The table includes subgroups within each variable, the number and percentage of participants in each subgroup, the mean scores for health literacy and GAD, and value of *p*s indicating statistical significance.

The results suggest that age had a statistically significant relationship with both health literacy and GAD, with participants aged over 60 having a higher mean health literacy score and lower mean GAD score compared to those aged less than 20 or between 20–40 years old. Gender had a statistically significant relationship with GAD, with men having a lower mean GAD score compared to women. Marital status was also found to have a statistically significant relationship with both health literacy and GAD, with married participants having a higher mean health literacy score and higher mean GAD score compared to single participants. Level of education had a statistically significant relationship with health literacy but not with GAD, with postgraduate participants having a higher mean health literacy score compared to those with school degrees or undergraduate degrees. Employment status had a statistically significant relationship with both health literacy and GAD, with retired participants having a higher mean health literacy score and lower mean GAD score compared to unemployed or employed participants. Homemakers had the highest mean health literacy score but the lowest mean GAD score among all employment subgroups.

Overall, the table provides valuable insights into the relationship between demographic variables and health literacy and GAD among the study population, highlighting the need for targeted interventions to address the unique needs of different subgroups.

Table 2 presents the results of the association between the mean (standard deviation) GAD score, 5.25 (3.16) out of a possible score of 12 with a range of scores between 1 and 12, and the mean (standard deviation) health literacy score, which was 52.91 (9.39) out of a possible score of 100 with a range of scores between 18 and 78, among individuals aged 18–60 in Khuzestan province. The table includes the mean scores and standard deviations for GAD and health literacy, the value of *p*, and the Pearson correlation coefficient.

**Table 2:** Association between mean generalized anxiety disorder score and health literacy.

Variables	Mean (SD)	Value of *p*	Pearson Correlation
Generalized anxiety disorder	5.25 (3.16)	0.0001	−0.17
Health literacy	52.91 (9.39)		

The results suggest that there is a statistically significant negative correlation between GAD and health literacy. Specifically, as health literacy increases, the mean GAD score decreases (*p* < 0.05). The Pearson correlation coefficient of −0.17 indicates a weak negative correlation between these two variables. Overall, the table provides valuable insights into the relationship between GAD and health literacy, highlighting the importance of addressing both variables in interventions aimed at improving mental health outcomes.

Table 3 includes subgroups based on the level of health literacy, the number and percentage of participants in each subgroup, mean GAD scores with 95% range, and value of ps indicating statistical significance. The results suggest that there is a statistically significant association between the level of health literacy and mean GAD scores. Participants with inadequate health literacy had the highest mean GAD score [5.8 (5.5–6.1)], followed by those with marginal health literacy [4.9 (4.7–5.2)]. Participants with adequate health literacy had the lowest mean GAD score [3.9 (3.1–4.6)]. The value of *p*s indicate statistical significance for all subgroups, suggesting that the association between health literacy and GAD is significant. The results suggest that lower levels of health literacy are associated with higher levels of GAD. Targeted interventions aimed at improving health literacy may be necessary to address the unique needs of individuals with inadequate or marginal health literacy and reduce the risk of GAD.

**Table 3:** Association between mean generalized anxiety disorder score and level of health literacy among individuals aged 18–60 years in Khuzestan province.

Variable	Subgroup	No. (%)	Mean (95%Range)	*p* value
Health literacy	Inadequate	427 (37.8)	5.8 (5.5–6.1)	0.001
	Marginal	628 (55.6)	4.9 (4.7–5.2)	0.01
	Adequate	75 (6.6)	3.9 (3.1–4.6)	0.02

Table 4 presents the results that suggest that age had a statistically significant relationship with health literacy among women, with participants aged 41–60 having the highest mean health literacy score compared to other age groups (p2 < 0.05). Furthermore, there was a statistically significant relationship between age and health literacy among men (p1 < 0.05).

**Table 4:** Association between the mean score of health literacy and demographic variables gender-wise in Khuzestan province.

Variable	Subgroup	Men *N* = 552	p1 value (Men)	Women *N* = 578	p2 value (Women)
Age	20<	51.4 (42.9–59.9)	0.001	51.4 (46.7–56.1)	0.02
	20–40	50.0 (48.7–51.3)		52.3 (51.2–53.3)	
	41–60	54.0 (53.0–55.0)		54.7 (53.5–55.8)	
	61<	55.7 (52.5–58.9)		52.7 (45.5–59.9)	
Marital Status	Single	49.4 (47.1–51.7)	0.01	53.2 (51.5–54.8)	0.9
	Married	53.1 (52.3–53.9)		53.2 (52.4–54.1)	
Education	Under high school	52.3 (50.5–54.0)	0.8	52.0 (50.1–53.8)	0.003
	Undergraduate	52.4 (48.7–56.1)		50.4 (47.4–53.4)	
	Postgraduate	52.9 (51.7–54.1)		55.3 (54.0–56.6)	
Employment	Unemployed	51.4 (47.7–55.0)	0.08	47.3 (42.9–51.7)	0.001
	Homemakers	49.0 (43.4–54.2)		52.9 (51.4–54.3)	
	Employer	52.4 (51.4–53.3)		53.6 (52.5–54.7)	
	Retirement	54.7 (53.0–56.5)		55.2 (53.3–57.1)	
	Others	50.5 (47.8–53.3)		53.7 (51.1–56.3)	

Married men had a higher mean health literacy score compared to single women. However, there was a statistically significant relationship between marital status and health literacy among men (p1 < 0.05).

Education had a statistically significant relationship with health literacy, with postgraduate women having a higher mean health literacy score compared to those with an undergraduate degree or under high school education (p2 < 0.05). However, there was no statistically significant relationship between education and health literacy among men.

Employment had a statistically significant relationship with health literacy among women, with unemployed women having the lowest mean health literacy score compared to other employment (p2 < 0.05), and there was no statistically significant relationship between employment and health literacy among men.

The results of Table 5 suggest that age had a statistically significant relationship with GAD among women (p2 < 0.05), with participants aged 20–40 having the highest mean GAD score compared to other age groups. However, there was no statistically significant relationship between age and GAD among men. Marital status had a statistically significant relationship with GAD, with married women having a higher mean GAD score compared to single women (p2 < 0.05). However, there was no statistically significant relationship between marital status and GAD among men.

**Table 5:** Association between the mean score of general anxiety disorder (GAD) and demographic variables gender-wise in Khuzestan province.

Variable	Subgroup	GAD in Men *N* = 552	P1 value (Men)	GAD in Women *N* = 578	P2 value (Women)
Age	20<	5.0 (2.7–7.4)	0.5	4.0 (2.9–5.1)	0.02
	20–40	5.2 (4.8–5.7)		5.7 (5.4–6.0)	
	41–60	4.8 (4.5–5.2)		5.2 (4.8–5.6)	
	61<	4.6 (3.5–5.7)		4.5 (2.1–6.9)	
Marital Status	Single	4.6 (4.0–5.3)	0.2	4.9 (4.5–5.4)	0.02
	Married	5.1 (4.8–5.4)		5.6 (5.3–5.9)	
Education	Under high school	5.4 (4.8–6.1)	0.1	5.5 (5.0–6.1)	0.4
	Undergraduate	5.1 (4.6–5.6)		5.9 (4.8–6.9)	
	Postgraduate	4.6 (4.2–5.0)		5.6 (5.1–6.1)	
Employment	Unemployed	7.3 (5.9–8.7)	0. 1	6.0 (4.8–7.2)	0.01
	Homemakers	8.0 (6.7–9.8)		5.9 (5.4–6.4)	
	Employer	4.9 (3.8–5.2)		5.4 (5.0–5.8)	
	Retirement	4.5 (3.8–5.2)		4.6 (3.7–5.5)	
	Others	5.2 (4.3–6.0)		4.7 (4.1–5.3)	

Education had no statistically significant relationship with GAD among women or men.

Employment had a statistically significant relationship with GAD among women (p2 < 0.05), with unemployed women having the highest mean GAD score compared to other employment subgroups. However, there was no statistically significant relationship between employment and GAD among men. Overall, the table provides insights into the association between demographic variables and GAD, specifically considering gender differences, in Khuzestan province. The results suggest that age, marital status, and employment may be factors influencing GAD among women. Targeted interventions may be necessary to address the unique needs of different subgroups based on these demographic variables.

## Discussion

Enhancing health literacy and promoting awareness regarding COVID-19, its transmission, and preventive measures can significantly contribute to the effective management of anxiety and stress induced by the virus. This is particularly crucial during periods of infectious disease crises and epidemics (Paakkari and Okan, 2020). It is important to note that anxiety can have adverse consequences on an individual’s personal and social well-being, diminishing their ability to cope with physical and mental health challenges and ultimately leading to a decline in their overall quality of life (Ryan and Twibell, 2000).

In this particular study, the researchers found that the average health literacy score among the participants was 9.52 ± 3.9. Additionally, the mean score (with standard deviation) for GAD resulting from fear of contracting COVID-19 was 2.5 ± 1.3. A significant negative correlation was observed between the mean GAD score and health literacy. Furthermore, a separate study focused on kidney patients revealed that the mean GAD score due to fear in this group was 13.3 ± 8.5, indicating a statistically significant difference across various levels of health literacy among the participants under investigation (Qobadi et al., 2014). These findings highlight the importance of health literacy in relation to anxiety levels among kidney patients.

Moreover, the results of this study demonstrated a significant negative correlation between health literacy and GAD in patients attending the Shahid Beheshti Polyclinic in Karaj (Shams et al., 2020). This suggests that individuals with higher levels of health literacy tend to have lower levels of anxiety related to their health.

Other research conducted by Alizadeh Aghdam et al. (2017) and Reavley et al. (2014) also revealed a significant positive association between health literacy and mental health. Similarly, Dodson et al. (2016) found that patients with higher health literacy exhibited fewer symptoms of depression and anxiety. These collective findings emphasize the importance of promoting health literacy as a means to reduce anxiety and improve mental well-being among individuals. By enhancing knowledge and understanding of health-related information, individuals can better manage their anxiety and take proactive steps toward maintaining their overall well-being.

According to the findings of Rabiaah’s study, a significant percentage of medical students, specifically 77%, experienced stress related to Middle East respiratory syndrome coronavirus (MERS-CoV). Among these students, 18.4% reported at least mild anxiety, while 6.4% reported moderate anxiety. Notably, no severe anxiety was observed among any of the students. The study also examined the average anxiety level resulting from fear among the participants, which was measured to be 3.1 ± 2.7. This suggests a moderate level of anxiety on average among the medical students due to their concerns about MERS-CoV (Al-Rabiaah et al., 2020).

Furthermore, the study revealed that female students had higher stress levels compared to their male counterparts. This indicates a gender difference in the experience of stress related to MERS-CoV among medical students. Additionally, the study identified a clear relationship between the stress levels of medical students and the presence of widespread anxiety disorder. This implies that higher stress levels among the students may contribute to the development or exacerbation of anxiety disorders.

These findings highlight the need for effective strategies to address and manage stress and anxiety among medical students, particularly during outbreaks of infectious diseases like MERS-CoV. Providing appropriate support systems and interventions can help mitigate the negative impact of stress and anxiety on the well-being and academic performance of medical students.

A study conducted by Lai et al. (2020) revealed that among healthcare workers providing services to COVID-19 patients, the level of anxiety was reported as 55.4% normal, 32.3% mild anxiety, 7% moderate anxiety, and 5.3% severe anxiety. It was found that women and individuals with direct contact with patients experienced higher levels of stress.

In a separate study from South Korea during the MERS-CoV outbreak, it was found that 7.6% of quarantined individuals exhibited signs of anxiety, while 16.6% experienced anger and irritability. However, after 6 months of quarantine, only 3% of individuals continued to experience anxiety, and anger and irritability were present in only 6.4% (Jeong et al., 2016).

According to the results of another study, 63.5% of MERS-CoV survivors suffered from severe neurological and psychological problems. Among these individuals, anxiety disorder was observed in 34.9%, and depression in 30.2%. It was noted that those who had lost loved ones during the epidemic, had experienced severe illness requiring mechanical ventilation, or had pre-existing anxiety disorders before the virus outbreak were more likely to experience these symptoms (Shin et al., 2019). These findings emphasize the significant impact of infectious disease outbreaks on mental health, particularly among healthcare workers and survivors. It highlights the need for appropriate support and interventions to address anxiety, stress, and other psychological challenges faced by individuals during such crises.

Individuals with higher levels of health literacy and awareness of health behaviors can play a crucial role in reducing mental and psychological illnesses and anxiety caused by diseases. By adhering to preventive measures and disease control, especially during crises, these individuals can effectively manage their health and well-being. Overall, promoting health literacy and awareness can have a positive impact on mental health outcomes, particularly during challenging times.

In the current study, the prevalence of inadequate, marginal, and adequate health literacy among the participants was found to be 8.37% (427 individuals), 6.55% (628 individuals), and 6.6% (75 individuals), respectively. A statistically significant difference was observed in the mean score of GAD among individuals with different levels of health literacy. Similarly, in a separate study focusing on kidney patients, it was found that 25% had inadequate health literacy, 8.9% had average health literacy, and 2.65% had adequate health literacy. There was also a statistically significant difference between different levels of health literacy and anxiety scores. These findings highlight the importance of health literacy in relation to anxiety levels among the subjects under investigation. Adequate health literacy appears to be associated with lower anxiety scores, emphasizing the need to promote and enhance health literacy in order to address and manage anxiety effectively (Alizadeh Aghdam et al., 2017).

This study found a statistically significant difference in the mean health literacy among men of different age groups and marital status, as well as among women of different age groups, educational levels, and occupational status. However, no statistically significant difference was observed in the health literacy of men with different educational and occupational statuses or women with different marital statuses. In another study, a statistically significant difference was observed between health literacy and educational levels. However, no statistically significant difference was found between health literacy and gender, age groups, marital status, or occupational status.

These findings suggest that demographic factors such as age, gender, educational level, marital status, and occupational status may influence health literacy levels. Therefore, it is important to consider these factors when developing interventions to improve health literacy and promote better health outcomes (Alizadeh Aghdam et al., 2017).

These results highlight the gender-specific differences in the relationship between demographic variables and anxiety disorders. It suggests that age, marital status, and occupation may play a role in anxiety levels among women, while educational level may not have a significant impact on anxiety disorders in women. Additionally, the study indicates that advanced medical education may be associated with lower stress and anxiety levels among students (Jeong et al., 2016). These findings contribute to a better understanding of the factors influencing anxiety disorders and can inform interventions and support strategies to address and manage anxiety effectively in different populations.

A study conducted in Iran revealed that there was no statistically significant difference in the level of anxiety and stress during the COVID-19 pandemic among individuals based on gender, age, and marital status. However, there was a statistically significant difference based on education level (Khademian et al., 2021). These results highlight the impact of education levels on anxiety and stress levels among the general population during the pandemic. They also emphasize the significant anxiety experienced by healthcare workers, particularly in relation to their gender, age, and job position. In another study, it was found that the mean score of anxiety among healthcare workers during the COVID-19 outbreak had the highest correlation with anxiety based on gender, age, and job position, and this correlation was statistically significant. Understanding these factors can help in developing targeted interventions and support systems to address and manage anxiety effectively, both among the general population and healthcare workers, during challenging times such as the COVID-19 pandemic (Kaveh et al., 2020).

A survey showed that 6.9% of teachers experienced significant anxiety during the COVID-19 pandemic. Their anxiety levels regarding the COVID-19 epidemic were found to be predictive of their age and awareness of future curriculum planning. Specifically, older teachers (aged 41–50) experienced higher levels of anxiety compared to others. However, there was no statistically significant relationship between gender and anxiety levels related to the disease (Ebrahimi, 2020). Based on these findings, it can be inferred that individuals with low health literacy in the population of Khuzestan may experience increased levels of stress, anxiety, and psychological symptoms during the COVID-19 pandemic. These results highlight the importance of addressing health literacy and providing appropriate support and interventions to mitigate the negative psychological impact of the pandemic on this population.

## Conclusion

The results of the study indicated that higher levels of health literacy were associated with reduced generalized anxiety disorders caused by the fear of contracting COVID-19. Therefore, promoting awareness and health literacy about the virus, its transmission, prevention methods, and management strategies can be highly effective in managing stress and anxiety related to the pandemic. By focusing on health literacy, addressing widespread anxiety disorders, and promoting preventive behaviors, health officials, planners, and policymakers can effectively promote health literacy and mitigate the negative psychological impact of the pandemic. Utilizing health services can also be an important tool in this effort. Overall, these findings highlight the importance of promoting health literacy as a key strategy to address anxiety and stress related to COVID-19 and to support individuals and communities in managing their health and well-being during this challenging time.

## Limitations of the study

1. Online self-report measures: The study may have relied on self-report measures for assessing health literacy and generalized anxiety disorder, which can be subject to recall bias or social desirability bias.
2. Cross-sectional design: If the study used a cross-sectional design, it may not capture changes in health literacy and generalized anxiety disorder over time, limiting the ability to establish causal relationships.
3. Contextual factors: The study’s findings may be specific to the Khuzestan province in Iran and may not be generalizable to other regions or populations with different sociocultural contexts.

## Data availability statement

The raw data supporting the conclusions of this article will be made available by the authors, without undue reservation.

## Ethics statement

The studies involving humans were approved by Ahvaz Jundishapur University of Medical Sciences, IR.AJUMS.REC.1399.089. The studies were conducted in accordance with the local legislation and institutional requirements. Written informed consent for participation in this study was provided by the participants.

## Author contributions

AD: Data curation, Software, Writing – original draft. NN: Formal analysis, Investigation, Writing – original draft. NH: Data curation, Project administration, Writing – original draft. MJ: Data curation, Project administration, Writing – original draft. SY: Data curation, Methodology, Writing – original draft. HM: Conceptualization, Writing – review & editing. MS: Conceptualization, Writing – original draft. MD: Software, Writing – review & editing. MC: Conceptualization, Supervision, Writing – review & editing.
